# Omega-3 fatty acids in mental disorders: from neurobiological and metabolic mechanisms to therapeutic potential

**DOI:** 10.3389/fnut.2026.1748196

**Published:** 2026-04-02

**Authors:** Katharina Fleig, Leoni Haslinger, Christine Dawczynski, Iris-Tatjana Kolassa

**Affiliations:** 1 Department of Psychosomatic Medicine and Psychotherapy, Ulm University Medical Center, Ulm, Germany; 2 Department of Clinical & Biological Psychology, Faculty of Engineering, Computer Science and Psychology, Ulm University, Ulm, Germany; 3 German Center for Mental Health (DZPG), Partner Site Mannheim – Heidelberg – Ulm, Ulm, Germany; 4 German Center for Child & Adolescent Health (DZKJ), Partner Site Ulm, Ulm, Germany; 5 Institute of Nutritional Sciences, Friedrich Schiller University Jena, Jena, Germany

**Keywords:** docosahexaenoic acid, eicosapentaenoic acid, fatty acid profile, mental disorders, nutritional psychiatry, omega-3 fatty acids, omega-6/omega-3 ratio

## Abstract

Nutritional psychiatry is an emerging field. Micro- and macro-nutrients play a role in energy metabolism and the regulation of inflammation; particularly, an insufficient dietary intake of omega-3 fatty acids and an imbalanced intake of omega-6/omega-3 fatty acids, with a shift toward increased inflammation, are of relevance for the pathophysiology of mental disorders. This review summarizes evidence on the role of omega-3 fatty acids in the pathophysiology of mental disorders (schizophrenia, affective and anxiety disorders, post-traumatic stress disorder, and eating disorders), neurodevelopmental disorders (attention-deficit/hyperactivity disorder and autism spectrum disorder) and neurodegenerative disorders (Alzheimer's disease) and explores potential treatment implications. In addition, the underlying neurobiological mechanisms through which omega-3 fatty acids might exert a protective effect are also discussed. Despite methodological variability and heterogeneous results, an increasing body of evidence suggests that omega-3 deficiency and altered fatty acid profiles are modifiable risk factors and potential biomarkers for mental disorders. The integration of omega-3 supplementation as an adjuvant to state-of-the-art therapy offers the potential for a low-risk intervention with meaningful clinical outcomes. However, clinical monitoring is recommended to avoid adverse effects and to adjust the dosage according to individual and disease-specific factors.

## 1 Introduction

### 1.1 Omega-3 fatty acids and mental health

Mental disorders currently rank among the leading contributors to the global burden of disease, posing substantial individual and societal challenges (1, 2). In parallel with rising rates of psychiatric disorders, industrialized societies have seen a shift toward higher dietary omega-6 to omega-3 ratios. Epidemiological studies suggest an inverse relationship between omega-3 intake or fish consumption and the prevalence of depressive disorders and cardiovascular diseases (3–5). Metabolic dysregulation within fatty acid metabolism may play a central role in the development of depressive disorders, which could also explain the high comorbidity rates (5).

Despite extensive research, the etiology of mental disorders remains only partially understood, and conventional treatment approaches, such as psychotherapy, pharmacotherapy, or a combination of both, often exhibit notable limitations (6). For instance, up to 30% of patients with depression do not respond to antidepressants, and many experience adverse effects or discontinue treatment due to poor tolerability, dependency concerns, or stigma (7, 8).

Given the modest efficacy of standard interventions, interest in well-tolerated, low-risk alternatives is increasing (9). One such candidate is omega-3 fatty acid supplementation, first proposed by Rudin (10) for potential benefits in both cardiovascular and mental health.

Numerous studies have reported altered fatty acid profiles and immunological abnormalities across a range of psychiatric disorders, including schizophrenia, depression, bipolar disorder, anxiety disorder, post-traumatic stress disorder (PTSD), attention-deficit/hyperactivity disorder (ADHD), autism spectrum disorders (ASD), and Alzheimer's disease (AD). However, findings remain inconsistent, possibly due to methodological heterogeneity (5).

This review provides a systematic and critical assessment of current scientific findings regarding the association between omega-3 fatty acids and a range of mental illnesses.

### 1.2 Classification of PUFA and fatty acid metabolism

**Figure 1:**
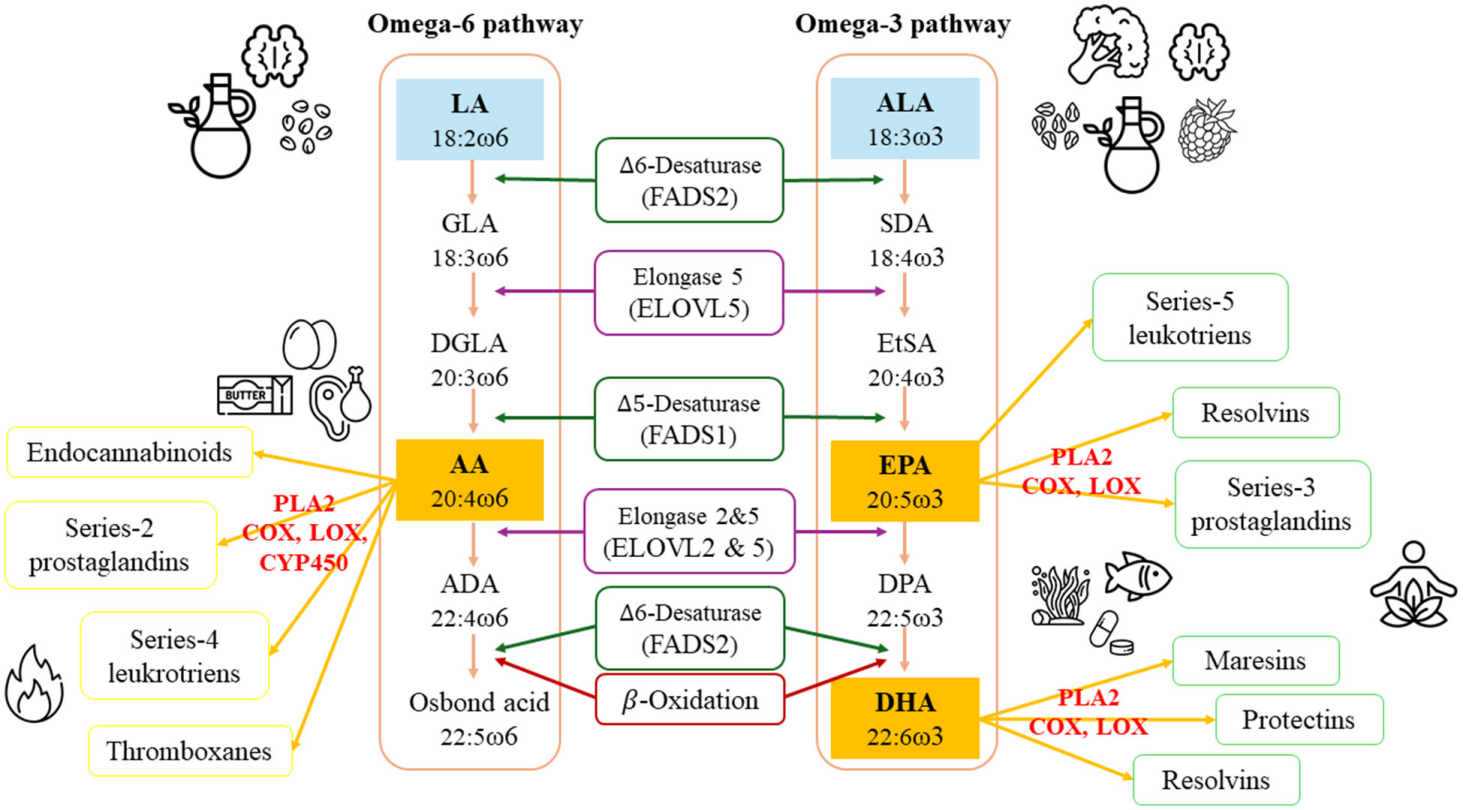
Fatty acid metabolism of omega-6 and omega-3 PUFA. LA, linoleic acid, 18:2ω6; GLA, gamma-linoleic acid, 18:3ω6; DGLA, dihomo-γ-linoleic acid, 20:3ω6; AA, arachidonic acid, 20:4ω6; ADA, adrenic acid, 22:4ω6; ALA, alpha-linolenacid,18:3ω3; SDA, stearidonic acid, 18:4ω3; EtSA, eicosatetraenoic acid, 20:4ω3; EPA, eicosapentaenoic acid, 20:5ω3; DPA, docosapentaenoic acid, 22:5ω3; DHA, docosahexaenoic acid, 22:6ω3; PLA2, phospholipase A2; COX, cyclooxygenase; LOX, lipoxygenase; CYP450, cytochrome P450 enzyme. Simplified overview of omega-6 and omega-3 fatty acid metabolism and their enzymatic competition. LA and ALA are obtained from plant-based foods and are subsequently converted into their long-chain derivatives—AA, EPA, and DHA—via a cascade of shared enzymatic steps. LA and ALA compete for the same desaturases and elongases. Metabolites derived from AA are predominantly pro-inflammatory eicosanoids, whereas those derived from EPA and DHA tend to exert anti-inflammatory effects.

Fatty acids are essential structural components of cell membranes, with polyunsaturated fatty acids (PUFAs) playing a particularly important role in maintaining membrane fluidity due to their multiple double bonds (11, 12). Figure 1 gives an overview of endogenous PUFA synthesis in the liver, starting from essential fatty acids linoleic acid (LA) and alpha-linolenic acid (ALA) (13, 14).

LA is a precursor to arachidonic acid (AA; 20:4ω6), which is also directly sourced from animal products like meat, butter, and eggs (11). Conversely, omega-3 PUFAs eicosapentaenoic acid (EPA; 20:5ω3), docosapentaenoic acid (DPA; 22:5ω3), and docosahexaenoic acid (DHA; 22:6ω3) are predominantly found in fatty marine fish and algae (15, 16). ALA, obtained from plant-based sources, for example, flaxseed and chia seed, can be converted endogenously into EPA and DHA through several desaturase and elongase steps (17). Although conversion rates of ALA to EPA and DHA are limited, with rates of 8% for EPA and 0.02%−4% for DHA, respectively (18), ALA serves as a precursor for long-chain omega-3 PUFAs and likely exerts independent biological effects. Evidence suggests that ALA may have a neuroprotective and anti-inflammatory potential concerning systemic, neuroinflammatory, and mental disorders (19).

PUFAs compete for the same set of desaturase and elongase enzymes during their metabolic conversion, most notably delta-6-desaturase [D6D; encoded by the gene fatty acid desaturase 2 (FADS2)] (15). Excessive intake of LA can inhibit the conversion of ALA into EPA and DHA due to enzymatic competition. Therefore, the dietary ratio of LA to ALA critically influences the efficiency of the synthesis of long-chain omega-3 derivatives (20). Although these enzymes show a higher binding affinity for n-3 substrates, this advantage is only effective under a favorable omega-6 to omega-3 ratio of approximately 1:1–4:1 (21, 22). However, typical Western diets, characterized by a high intake of processed foods, tend to produce a skewed ratio of 10–20:1 in favor of n-6 fatty acids, promoting AA synthesis. From an evolutionary perspective, a ratio of 1–4:1 is considered optimal for maintaining health (23, 24).

The metabolic end products EPA, DPA, DHA, and AA exist either in esterified form within complex lipids or as free (unesterified) fatty acids (13). Once incorporated into cell membranes, PUFAs influence membrane fluidity and structural organization (11). In response to physiological or pathological stimuli, they are released from the sn-2 position of membrane phospholipids via the action of phospholipase A2 (PLA2). The liberated free fatty acids are subsequently metabolized by cyclooxygenase (COX), lipoxygenase (LOX), and cytochrome P450 (CYP450) enzymes into a variety of bioactive derivatives, known as eicosanoids, which play key roles in the regulation of inflammatory processes (22). Eicosanoids derived from AA are predominantly pro-inflammatory, whereas those synthesized from EPA exhibit anti-inflammatory potential (21). While a moderate level of AA-derived eicosanoid production is essential for maintaining physiological homeostasis, chronically elevated AA availability—particularly in the context of insufficient omega-3 intake—may contribute to the development of systemic inflammatory processes (20).

### 1.3 Biological effects of EPA, DPA, and DHA on mental and physical health

EPA, DPA, and DHA exert a wide range of biological effects, including anti-arrhythmic, antithrombotic, and endothelium-modulating actions (16, 20). Chronic low-grade inflammation is a hallmark of cardiometabolic diseases, and it has also been observed across various psychiatric and neurodegenerative disorders, including depression, bipolar disorder, schizophrenia, anxiety disorders, ADHD, ASD, and AD (25–27). Matits et al. (28) reported transdiagnostic elevations of interleukin-6 (IL-6) and C-reactive protein (CRP) in depression, bipolar disorder, schizophrenia, generalized anxiety disorder, and PTSD. These changes, however, typically affect only a subset of patients and remain within the range of low-grade inflammation (28, 29). The anti-inflammatory properties of EPA and DHA may help explain why individuals with elevated inflammatory markers appear to benefit most from omega-3 supplementation (20).

In the gray matter of the brain and in the retina, DHA is the predominant structural fatty acid (30). DHA constitutes about 10%−20% of the total fatty acids in the brain, and up to 40% of the PUFA in neuronal membranes (31–33). It plays a crucial role in key neuronal processes, such as neurotransmission, signal transduction, and synaptic plasticity (20). In addition, DHA exerts effects through the regulation of gene expression, stabilization of cell membranes, and protective actions against apoptosis (34). DHA is enzymatically converted into specialized pro-resolving mediators (SPMs), including resolvins, protectins, and maresins. These mediators modulate neuronal metabolism by reducing oxidative stress and influencing cerebral immune responses (35). Protectins are primarily produced under cellular stress, including oxidative stress and early neurodegeneration, promoting neuronal survival. Resolvins facilitate the resolution of ongoing neuroinflammation and contribute to neurogenesis. Maresins, primarily synthesized by macrophages, enhance tissue repair and regenerative processes (Figure 1) (35).

In contrast to DHA, EPA is only present in small amounts in neuronal membranes but demonstrates significant anti-inflammatory and antidepressant effects (36).

DPA is the second omega-3 long-chain PUFA found in the brain, with a cerebral concentration about 70 times lower than that of DHA. It occurs as an intermediate in the metabolism between EPA and DHA (37). DPA can be retro-converted back to EPA; therefore, the conversion from DPA to DHA is limited (38). DPA and its metabolites seem to have anti-inflammatory and further beneficial health effects, such as the improvement of cellular plasticity and platelet aggregation. Since dietary DPA appears to be a good source of EPA, it could help to increase the omega-3 status (37).

Building on the fundamental biological roles of long-chain omega-3 PUFA, such as EPA and DHA, a growing body of evidence has investigated their relevance across various psychiatric conditions. The following section considers both studies comparing fatty acid composition in patients and healthy controls, as well as interventional supplementation trials, to provide a comprehensive overview of the scientific evidence regarding the relationship between omega-3 fatty acids and psychiatric disorders.

## 2 Methods

### 2.1 Search strategy

A narrative literature review was conducted to summarize current evidence on the role of omega-3 fatty acids in several mental disorders (schizophrenia, affective and anxiety disorders, PTSD, and eating disorders), neurodevelopment disorders (ADHD and ASD), and neurodegenerative disorders (AD). Relevant studies were identified through searches of PubMed for articles published in English up to 2025, using the keywords “omega-3 fatty acids,” “EPA,” “DHA,” “mental disorders,” “psychiatric disorders,” “schizophrenia,” “affective disorder,” “depression,” “bipolar disorder,” “anxiety disorder,” “PTSD,” “eating disorder,” “ADHD,” “ASD,” “AD,” and related terms.

### 2.2 Study selection

Studies were selected based on PUFA-status in the respective disorders and on evidence regarding the preventive and therapeutic potential of omega-3 PUFA supplementation.

### 2.3 Study types

The materials for this narrative review consisted of peer-reviewed journal articles, including clinical studies, animal studies, Mendelian randomization (MR) studies, case series, meta-analyses, systematic reviews and narrative reviews, retrieved from “PubMed.” No primary data or experimental samples were collected for this study.

## 3 Disorder-specific findings

**Figure 2:**
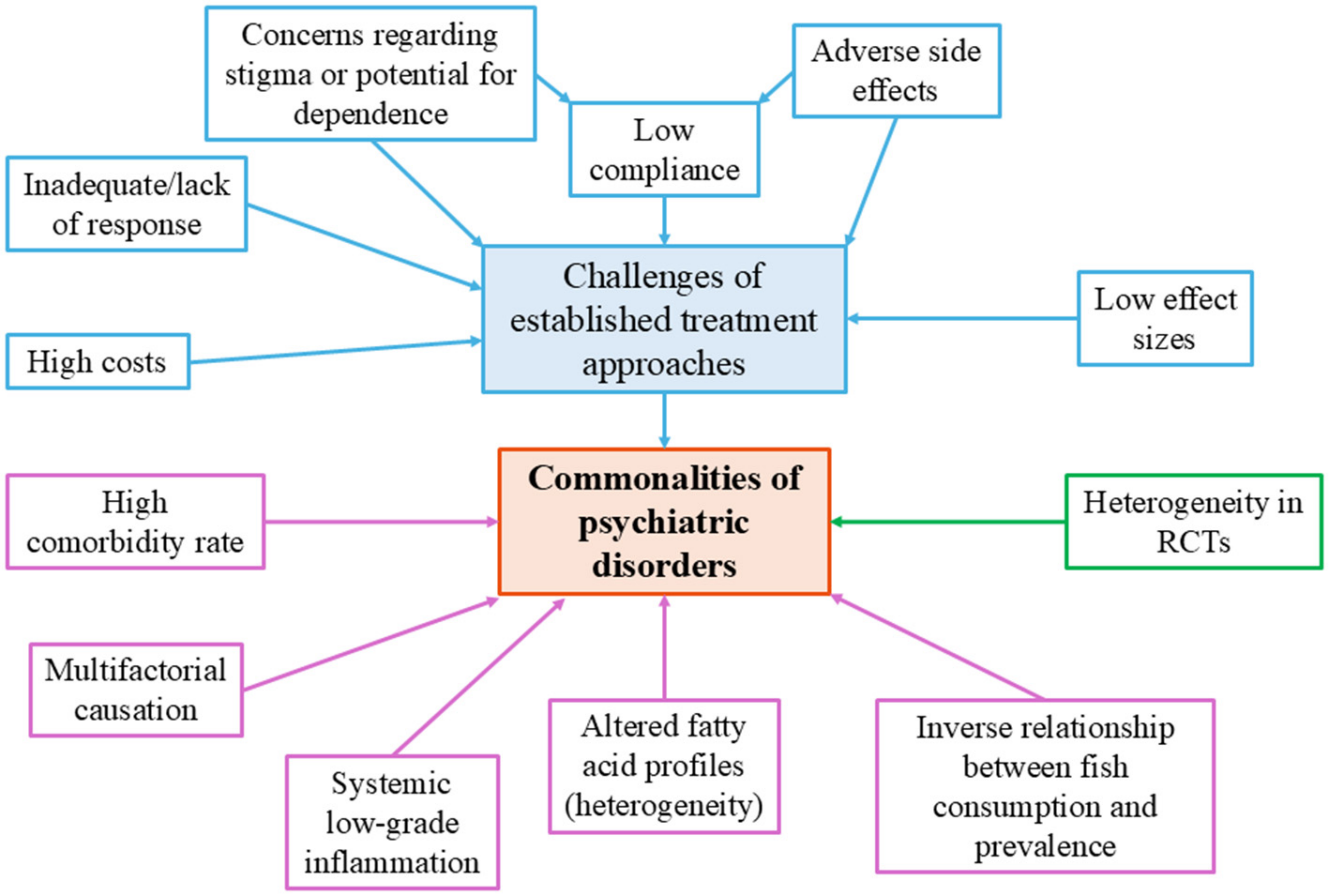
Transdiagnostic commonalities of mental disorders. The figure summarizes shared challenges of psychiatric disorders and their treatments. Limitations of current approaches (e.g., low compliance, high costs, side effects) contribute to transdiagnostic features such as high comorbidity, inflammation, altered fatty acid profiles, and multifactorial causation. Additional factors include RCT heterogeneity and the inverse association between fish consumption and disorder prevalence.

Findings from randomized controlled trials (RCTs) on omega-3 supplementation are heterogeneous both across and within psychiatric diagnoses, making it difficult to issue a universal recommendation for its preventive or therapeutic use (39). Figure 2 summarizes transdiagnostic commonalities across various psychiatric disorders. However, given the existence of neutral or negative findings (40), a differentiated, disorder-specific evaluation of its clinical utility is warranted. The following section provides such an analysis.

### 3.1 Schizophrenia

#### 3.1.1 PUFA-status

Based on the membrane phospholipid hypothesis, which posits that fatty acid metabolism is altered in schizophrenia (41), several studies have investigated the fatty acid status of individuals with schizophrenia in comparison to healthy controls. As summarized in Table 1, the findings indicate significant deficiencies in LA, AA, DHA, and total omega-3 PUFA. A meta-analysis of Hoen et al. (42) identified 18 studies comparing PUFA status in erythrocyte cell membranes between patients with schizophrenia and controls. They showed decreased levels of DHA in red blood cell membranes of patients with a standard mean difference (*SMD* = 0.61; *p* < 0.001), decreased levels of DPA (*SMD* = 1.14; *p* < 0.001), decreased levels of LA (*SMD* = 0.73; *p* < 0.001), and decreased levels of AA (*SMD* = 0.83; *p* < 0.001). Sethom et al. (43) further showed a significantly higher omega-6/omega-3 ratio in patients with schizophrenia compared to controls. However, the overall body of evidence remains heterogeneous, likely due to variations in study design, patient sampling, and numerous potential individual confounding factors (43, 44). Several studies report significantly lower DHA and AA levels in unmedicated patients compared to those receiving antipsychotic treatment (45–47). Particularly atypical antipsychotics may contribute to increased PUFA levels, potentially due to their antioxidant properties (43, 48). Sample heterogeneity also contributes to the variability in findings. For instance, Doris et al. (49) included 40 patients with a wide range of illness severity and 40 age- and sex-matched controls, and they found no significant differences in DHA and AA levels. In contrast, studies involving acutely ill patients tend to reflect the expected PUFA deficiency pattern in red blood cells by gas chromatography (50), whereas investigations in remitted individuals have reported an inverse profile (44). Furthermore, a separate analysis suggests the existence of sex-specific differences, with men exhibiting more pronounced DHA deficiencies compared to women (51). Three studies further identified a bimodal distribution of DHA and AA, suggesting the existence of two distinct subgroups (50, 52, 53): one group with high PUFA concentrations and another with low PUFA concentrations (44). Overall, the findings suggest that PUFA metabolism differs markedly from healthy controls in a subset of individuals with schizophrenia and may represent a modifiable risk factor (54).

**Table 1:** Fatty acid composition in schizophrenia.

Study	Characteristics	DHA	Total n3	LA	AA	n6/n3 ratio
Meta-analyses						
Hoen et al. (42)^*^	M; NM	↓ M, NM		↓ M	↓ M, NM	
Van der Kemp et al. (166)^**^	M; NM	↓ M, NM			↓ M (atypical	
					antipsychotics), NM	
Studies from meta-analyses						
Vaddadi et al. (175)^*, **^	ERY; treatment resistance	↑		↓	↓	
Vaddadi et al. (176)^**^	ERY; M	↓			↓	
Yao et al. (177)^*, **^	ERY; only men; M, NM	↓		↓	↓	
Peet et al. (53)^*^	ERY; M	↓		↓	↓	
Doris et al. (49)^*^	ERY	=			=	
Assies et al. (159)^*, **^	ERY; M	↓	↓		=	↑ AA/DHA
Khan et al. (47)^*^	ERY; M, NM	↓		↓	↓	
Khan et al. (47)^*^	ERY; NM vs. M	↓			↓	
Yao et al. (178)^*, **^	ERY; NM	=			↓	
Ranjekar et al. (179)^*, **^	ERY; only men; M	↓	↓		=	
Arvindakshan et al. (45)^*, **^	ERY; M, NM	↓ M, NM	↓ NM		↓ NM	
Arvindakshan et al. (45)^*, **^	ERY; NM vs. M	↓	↓		↓	
Evans et al. (46)^*, **^	ERY; NM	↓			=	
Evans et al. (46)^*, **^	ERY; M	=			=	
Evans et al. (46)^*, **^	NM vs. M	↓ NM			=	
Reddy et al. (160)^*, **^	ERY; NM	↓			↓	
Peet et al. (180)^*^	ERY; NM	↑	↑		=	↓ n6/n3
Yao et al. (181)^**^	ERY; NM	=			↓	
Kemperman et al. (182)^*, **^	ERY; M	↓	↓		↓	
Sumiyoshi et al. (183)^*, **^	ERY; typical antipsychotics	↓ M	↓ M		↓ M	
	M; NM	= NM	= NM		= NM	
Kale et al. (184)^*, **^	ERY, Liquor, PLA; NM	↓ ERY			= ERY	
		↑ Liquor			↓ Liquor	
		= PLA			= PLA	
Sethom et al. (43)^*, **^	ERY; only men; NM	↓	↓		↓	↑ n6/n3
Sethom et al. (43)^*, **^	ERY; only men; M	=	=		=	=
Messamore et al. (185)^*^	ERY; M				=	
Bentsen et al. (50)^*^	ERY; M	↓	↓		↓	↑ n6/n3
Additional studies						
Horrobin et al. (186)	PLA; patients vs. controls in 3 different states: England, Ireland and Scotland	↑ England and Ireland	↑ England	↓	↓ Scotland, England	↓ n6/n3
Horrobin et al. (187)	Postmortem frontal cortex	↑			↓	
Kaiya et al. (188)	PLA	=		↓	=	
Glen et al. (52)	ERY, PLA; patients with predominantly negative vs. positive symptoms	↓ ERY			↓ ERY	
		↑ PLA			= PLA	
Laugharne et al. (189)	ERY; M	↓			↓	
Peet et al. (190)	First-degree relatives of patients			↓		
Mahadik et al. (191)	Skin fibroblasts; NM	↓			=	
Yao et al. (192)	Postmortem caudate region	=		↓	↓	
McNamara et al. (51)	Postmortem OFC	↓			↓	↑ AA/DHA
Kim et al. (193)	ERY; UHR; patients with predominantly negative vs. positive symptoms	=	↓		=	↑ n6/n3
Medema et al. (44)	ERY; M; patients and siblings vs. controls	↑			↑	=

^*^Studies from Hoen et al. (42).

^**^Studies from van der Kemp et al. (166); empty fields: not reported; ↑ increased in schizophrenia; ↓ decreased in schizophrenia; = no significant difference; comparisons were—unless otherwise noted—conducted between individuals with schizophrenia and healthy controls.

DHA, docosahexaenoic acid, 22:6ω3; AA, arachidonic acid, 20:4ω6; LA, linoleic acid, 18:2ω6; ERY, erythrocytes; PLA, plasma; OFC, orbitofrontal cortex; UHR, ultra-high-risk population; M, on medication; NM, no medication; n6/n3, omega-6/omega-3 ratio.

#### 3.1.2 Preventive potential of omega-3 PUFA supplementation

So far, in one randomized, placebo-controlled trial in individuals at ultra-high risk of psychotic disorder aged 13–25 years, administration of a combination of DHA (480–560 mg/day) and EPA (700–840 mg/day) over 12 weeks was associated with reductions in psychotic symptom severity and a substantially lower transition rate to full-threshold psychosis compared with placebo. By 12 months, progression to psychosis occurred in only 4.9% of participants receiving omega-3 PUFA vs. 27.5% in the placebo group (*p* = 0.007, accompanied by improvements in positive (*p* = 0.010), negative (*p* = 0.020), and general symptoms (*p* = 0.010) as well as overall functioning (*p* = 0.002). The authors suggested that long-chain omega-3 PUFA supplementation might reduce the risk of progression from subthreshold psychotic symptoms to manifest psychotic disorder, and they proposed it as a safe preventive strategy in young individuals at elevated clinical risk (55).

#### 3.1.3 Therapeutic potential of omega-3 PUFA supplementation

Therapeutic intervention studies on omega-3 supplementation at different stages of schizophrenia, from first-episode psychosis to chronic schizophrenia, have yielded inconsistent findings, largely due to methodological differences and study limitations (56–59).

In a meta-analysis of 2021 for patients with a first episode of psychosis (mean age = 30.0 years), EPA-dominant PUFA supplementation at doses exceeding 2 g/day (mean duration of intervention = 17.7 weeks) was associated with improvements in total Positive and Negative Syndrome Scale scores (*p* < 0.001), positive (*p* < 0.001), and negative (*p* = 0.002) scale scores, and the general psychopathology subscale (*p* < 0.001) (58). First episode psychosis typically emerges in young adulthood, a phase of intensive neural maturation (58). Bozzatello et al. (56) suggested that supplementation at this stage could be more effective than during later chronic phases of schizophrenia, although evidence is limited and heterogenous because of small sample sizes, varying PUFA preparations and doses, and short intervention studies (56).

Ross et al. (59) raised doubts regarding the clinical efficacy of PUFA supplementation in established schizophrenia, reviewing five different RCTs administering 1–4 g of EPA in patients with schizophrenia (mean ages ranging from 37 to 45 years). Across these studies, treatment effects on psychotic symptoms were inconsistent and generally modest, supporting the view that omega-3 PUFA supplementation provides limited clinically relevant benefit in chronic schizophrenia. In contrast, more recent meta-analyses by Bozzatello et al. (56) and Goh et al. (58) reported promising results: supplementation with n-3 PUFA in chronic schizophrenic patients has been associated with modest reductions in overall symptom severity, including improvements in total PANSS scores (*p* = 0.003) and the general psychopathology subscale (*p* = 0.05) (58). In general, PUFA supplementation was reported to be safe and well-tolerated. Formulations providing higher doses of EPA (≥1 g/day) have been proposed as a potential adjunctive treatment, particularly in patients with more severe symptomatology (58). When administered alongside antipsychotic treatment, omega-3 supplementation may enhance medication response, reduce the required antipsychotic doses, and lower the risk of extrapyramidal side effects (60). However, the typically short supplementation periods (4–16 weeks) limit definitive conclusions regarding long-term efficacy (61).

#### 3.1.4 Conclusion

Overall, the existing evidence supports consideration of EPA-dominant supplementation (≥1 g/day) as an adjunctive treatment for patients with first-episode or chronic schizophrenia. Early initiation appears particularly important to correct pre-existing omega-3 deficiencies. Individuals with more severe symptoms and low baseline omega-3 status seem to benefit the most from such interventions (58). However, a recent guideline from the World Federation of Societies of Biological Psychiatry (WFSBP) and the Canadian Network for Mood and Anxiety Treatments (CANMAT) Taskforce does not currently recommend omega-3 PUFA supplementation at doses of 1–2 g/day for either adjunctive or monotherapy in the treatment of schizophrenia (Evidence grade A) (62).

### 3.2 Affective disorders

#### 3.2.1 PUFA-status in depressive and bipolar disorders

Numerous studies have reported significantly lower omega-3 levels, particularly DHA, as well as an increased omega-6/omega-3 ratio in individuals with depression and bipolar disorder. Exceptions are the studies by Fehily et al. (63) and Ellis and Sanders (64), who found an atypical fatty acid profile in depressed patients, showing elevated DHA and EPA levels. Interestingly, in a sample of 60 patients with major depressive disorder, Cussotto et al. (65) found that PUFA baseline status differed between responders and non-responders to standard antidepressants. Non-responders had lower omega-3 PUFA levels and a higher omega-6/omega-3 ratio compared to responders. There was also an association between DHA status and clinical treatment response, suggesting that DHA may serve as a predictive biomarker of treatment efficacy (65). The findings from studies on depression are summarized in Table 2.

**Table 2:** Fatty acid composition in depression.

Study	Characteristics	DHA	EPA	Total n3	LA	AA	n6/n3 ratio
Meta-analysis							
Lin et al. (194)		↓	↓	↓		=	
Studies from the meta-analysis							
Fehily et al. (63)	ERY; bipolar and unipolar depression	↑	↑		↓	=	
Maes et al. (195)	Serum	=	=	↓	=	=	↑ n6/n3
Edwards (196)	ERY	↓	↓	↓	=	=	
Peet et al. (197)	ERY	↓	=	↓	↓	=	=
Maes et al. (164)	PLA	=	↓	=	=	=	↑ AA/EPA
De Vriese et al. (198)	Serum; postpartum depression	↓	=	↓	=	=	↑ n6/n3
Tiemeier et al. (199)	PLA; older adults	↓	=	↓	=	↑	↑ n6/n3
Frasure-Smith et al. (200)	PLA; ACS	↓	=	↓	=	=	↑ n6/n3
Amin et al. (201)	ERY; ACS	↓	=	↓	=	=	
Aupperle et al. (202)	ERY; MS	=	=	=	=	=	
Féart et al. (203)	PLA; older adults	=	↓	=	=	=	=
Dinan et al. (204)	PLA	=	=			↑	↑ AA/EPA (only in non-responders on antidepressive M)
Rees et al. (205)	PLA; pregnancy	↓	=	↓		=	↑ n6/n3
Additional studies							
Ellis and Sanders (64)	PLA	↑	↑			=	
Mamalakis et al. (206)	Adipose tissue; mild depression	↓	=	=	=	=	=
Mamalakis et al. (207)	Adipose tissue; adolescents	=	=	=	=	=	=
Mamalakis et al. (207)	Adipose tissue; elderly individuals	=	=	=	=	=	↑ n6/n3
Parker et al. (208)	PLA; ACS	↓	=	↓			
McNamara et al. (209)	Postmortem OFC	↓					↑ AA/DHA (only women)
Schins et al. (210)	Serum; post-MI				=		↑ AA/EPA
Riemer et al. (211)	Serum	↓	=	↓		=	↑ n6/n3
McNamara et al. (212)	ERY	↓	=	↓	↓	=	↑ AA/DHA
Assies et al. (213)	Recurrent MDD	= PLA	= PLA	= PLA	↑ PLA	= PLA	= PLA
		↓ ERY	= ERY	↓ ERY	= ERY	↓ ERY	↑ n6/n3 ERY
Sublette et al. (214)	PLA	↓					
Rizzo et al. (215)	ERY; elderly women		↓				↑ AA/EPA
Pottala et al. (216)	ERY; adolescents	↓	=		↓	=	
Liu et al. (89)	PLA	↓	↓				↑ AA/EPA
Liu et al. (89)	MDD with vs. without anxiety disorder	↓	↓				↑ AA/EPA
McNamara et al. (217)	ERY; treatment-resistant adolescents	↓	=	↓	=	=	↑ AA/DHA
Otoki et al. (218)	PLA; seasonal MDD; summer vs. winter			↓ summer			
Thesing et al. (84)	PLA	↓		↓			
Cussotto et al. (65)	ERY; non-responders vs. responders on antidepressive medication	↓	↓	↓			↑ n6/n3

Empty fields: not reported; ↑ increased in depression; ↓ decreased in depression; = no significant difference; comparisons were—unless otherwise noted—conducted between individuals with depression and healthy controls.

DHA, docosahexaenoic acid, 22:6ω3; EPA, eicosapentaenoic acid, 20:5ω3; LA, linoleic acid, 18:2ω6; AA, arachidonic acid, 20:4ω6; ERY, erythrocytes; PLA, plasma; OFC, orbitofrontal cortex; ACS, acute coronary syndrome; MI, myocardial infarction; MS, multiple sclerosis; MDD, major depression; n6/n3, omega-6/omega-3 ratio.

Compared to unipolar depression, fewer studies have investigated PUFA composition in bipolar disorder. While some studies found no differences in fatty acid profiles between affected individuals and healthy controls, other findings confirm the pattern observed in depression, indicating an omega-3 deficiency (see Table 3). Studies also show significant differences in the omega-3 index between healthy and affected individuals, as well as an inverse association with symptom severity (66). In addition, in affective disorders, both the AA/EPA and the omega-6/omega-3 ratios were positively correlated with symptom severity, whereas EPA concentrations showed a negative correlation (67, 68).

**Table 3:** Fatty acid composition in bipolar disorder.

Study	Characteristics	DHA	EPA	Total n3	LA	AA	n6/n3 ratio
Meta-analyses							
McNamara and Welge (165)		↓	=		=	=	
Studies from meta-analyses							
Chiu et al. (219)	ERY	↓	=	=	=	↓	=
Ranjekar et al. (179)	ERY	=	↓	↓		=	
Clayton et al. (80)	ERY; children and adolescents	=	=	=		=	
McNamara et al. (212)	ERY	↓	=	↓	=	=	↑ n6/n3
McNamara et al. (220)	ERY	↓	=	↓	=	=	↑ n6/n3
Ross et al. (221)	ERY	=	=		=	=	
Additional studies							
Sublette et al. (222)	PLA	=	=	=		=	=
McNamara et al. (223)	Postmortem OFC	↓				↓	
Igarashi et al. (224)	Postmortem PFC	=	=	=	↑	=	=
Pomponi et al. (225)	PLA	↓	↑		↑	↑	
Evans et al. (226)	PLA	=	↓		↓	=	
Saunders et al. (81)	PLA	=	=		=	=	=
Koga et al. (227)	PLA	↓	↓		↑	↑	↑ AA/EPA
Ashizawa et al. (228)	PLA	=	=		↓	↓	

Empty fields: not reported; ↑ increased in BD; ↓ decreased in BD; = no significant difference; comparisons were conducted between individuals with BD and healthy controls.

DHA, docosahexaenoic acid, 22:6ω3; EPA, eicosapentaenoic acid, 20:5ω3; LA, linoleic acid, 18:2ω6; AA, arachidonic acid, 20:4ω6; ERY, erythrocytes; PLA, plasma; OFC, orbitofrontal cortex; PFC, prefrontal cortex; BD, bipolar disorder; n6/n3, omega-6/omega-3 ratio.

#### 3.2.2 Preventive potential of supplementation with omega-3 PUFA in depressive disorder

As a consequence, it can be assumed that the intake of omega-3 fatty acids may have a preventive effect on the development of depressive symptoms and disorders. To investigate this, a meta-analysis of 31 observational studies including 255,076 participants examined the association between fish consumption (*n* = 21) or dietary omega-3 PUFA intake (*n* = 15) and depression (69). Highest fish consumption in relation to the lowest was associated with a significant reduction in depression risk [RR = 0.78, 95% CI: (0.69–0.89)], while higher consumption of total omega-3 PUFA also reduced risk [EPA + DHA; RR = 0.82, 95% CI: (0.73–0.92)]. Dose-response analysis revealed a maximal benefit at approximately 1.8 g/day of omega-3 PUFA [RR = 0.30, 95% CI: (0.09–0.98)] (69). When evaluating this meta-analysis, however, it must be taken into account that studies of varying quality were included, among them observational studies and cross-sectional studies, which do not allow for causal interpretation (69).

#### 3.2.3 Therapeutic potential of supplementation with omega-3 PUFA in depressive disorder

Building on these preventive findings, an umbrella review has summarized the evidence of 22 meta-analyses that were published between 2007 and 2021 with sample sizes varying between 201 and 10,297 participants. There, the effects of omega-3 PUFA supplementation (EPA, DHA, or their combination) on depressive symptoms (duration of intervention: 4–160 weeks) were evaluated. The included meta-analyses were of high methodological quality (mean AMSTAR score = 10) with considerable heterogeneity (*I*^2^ >50% in 14 studies, including seven with *I*^2^ >75%). Sixteen of the 26 effect sizes demonstrated significant improvements in depressive symptoms, predominantly with small effect sizes, while five meta-analyses reported moderate to large effects (*SMD* ranging from −0.61 to −0.94). The other meta-analyses could not show a significant effect of omega-3 PUFA supplementation (70). Antidepressant effects were pronounced in individuals with major depressive disorder, with results pointing to a positive correlation between omega-3 PUFA dosage and symptom improvement. Furthermore, it is described that certain subgroups may benefit particularly from supplementation with long-chain omega-3 PUFA, including individuals with perinatal depression, inflammatory conditions, cardiovascular comorbidities, children and adolescents, as well as those with low baseline EPA and DHA levels and severe symptoms (70–73). EPA appeared to exert stronger antidepressant effects than DHA; nonetheless, omega-3 PUFA were not superior to conventional antidepressant medication (70). Overall, growing evidence points to clinically relevant benefits of omega-3 PUFA as a supportive adjunct therapy in addition to established treatments, particularly with EPA-dominant formulations (70, 74, 75).

Supplementation with 1–2 g/day of omega-3 PUFA, either only EPA or a formulation of predominantly EPA, over a period of at least 8 weeks, especially as an adjunct to standard treatments, has shown promising results in reducing depressive symptoms (70, 74–77). This recommendation is now reflected in international clinical guidelines for the evidence-based use of omega-3 PUFAs in psychiatry (62, 72). The International Society for Nutritional Psychiatry recommends the administration of omega-3 PUFA in MDD for “pregnant women, children, and the elderly, and prevention in high-risk populations” (72). Furthermore, they advise additionally monitoring potential side effects, such as gastrointestinal or dermatological symptoms (72). Another recent guideline by the WFSBP and the CANMAT Taskforce provides Level A evidence supporting the use of 1–2 g/day of EPA as an adjunctive treatment for major depressive disorder, though not as monotherapy (62). Especially in individuals with increased inflammation markers, obesity or malnutrition, an adjunct use of up to 4 g/day is recommended. Before surgery and when taking anticoagulants, administration should only be given with caution (62).

#### 3.2.4 Therapeutic potential of supplementation with omega-3 PUFA in bipolar disorder

Meta-analytic evidence indicates that adjunctive omega-3 PUFA therapy may improve depressive but not manic symptoms in bipolar patients (74, 78). Specifically, a meta-analysis of five RCTs including 291 participants with bipolar disorder examined adjunctive omega-3 PUFA supplementation (DHA and EPA) for a minimum of 4 weeks and found a significant moderate effect on bipolar depressive symptoms (Hedges' *g* = 0.34, *p* = 0.029; *I*^2^ = 30%), whereas no significant effect on manic symptoms was observed (Hedges' *g* = 0.20, *p* = 0.099; *I*^2^ = 0%) (78). Further meta-analytic evidence from three RCTs showed that adjunctive omega-3 PUFA supplementation (1–6.2 g EPA; 2–3.4 g DHA) in individuals diagnosed with bipolar disorder over 12–16 weeks significantly reduced depressive symptoms (*SMD*: 0.74, 95% CI: 0.38–1.10; *I*^2^ = 9%), whereas effects on mania were not significant, highlighting the potential therapeutic benefit of omega-3 as an add-on treatment in bipolar depression (74). However, methodological heterogeneity, small samples, and missing dietary data limit these findings. Current evidence is inconsistent, preventing clear treatment recommendations (78, 79). Some open-label studies report symptom improvements (80, 81), with greater therapeutic benefit when omega-3 PUFA are combined with pharmacotherapy or used in deficient patients (79, 81). The Clinical guidelines by Sarris et al. (62) weakly recommend 1–2 g of EPA for adjunct use to standard treatment.

#### 3.2.5 Results from MR studies on affective disorders

Observational studies are often limited by confounding factors and cannot definitively establish causal relationships between fatty acids and affective disorders (82). MR studies have been increasingly used to assess causality. They use genetic variants as natural instruments to investigate causal relationships between risk factors and diseases while minimizing confounders and reverse causality. In an MR study, Milaneschi et al. (83) found no significant evidence of a shared genetic risk between omega-3 PUFA and depression. Moreover, they did not demonstrate evidence supporting a causal effect of PUFA on major depression, nor of major depression on omega-3 PUFA levels. Similarly, a longitudinal study by Thesing et al. (84) found no uni- or bidirectional causal links, further arguing against causality. Nevertheless, these findings do not necessarily imply the absence of causality, particularly when considering the effects of targeted interventions or achieving optimal blood concentration.

PUFA may play a causal role in specific depression subtypes (83). Additional MR studies suggest more nuanced effects: genetically predicted higher omega-3 levels, particularly EPA, are associated with a reduced risk of major depression (85). Lu et al. (86) report that higher circulating omega-3 fatty acids correlate with a lower risk of bipolar disorder. Zeng et al. (82) identified a causal relationship between ALA and EPA and depression risk; EPA appeared protective, whereas ALA may act as a risk factor. Further evidence indicates that omega-3 fatty acids and the omega-6/omega-3 ratio could play an etiological role in bipolar disorder development in Europe (87).

#### 3.2.6 Conclusion

Overall, the current evidence suggests an association between low PUFA status and affective disorders, particularly major depression. However, findings in bipolar disorder are less consistent. EPA-dominant supplementation appears to most effectively alleviate depressive symptoms, while its impact on manic episodes is limited. Although MR studies have not demonstrated a clear causal relationship, subtype-specific or baseline-dependent effects remain plausible. Current evidence and international guidelines support the use of 1–2 g/day of EPA-dominant omega-3 PUFA as an adjunctive treatment in major depressive disorder, particularly in individuals from certain risk groups, and weakly in bipolar depression (62). Future research should focus on personalized, biomarker-guided approaches to better understand the mechanistic pathways and therapeutic potential of omega-3 PUFAs in mood disorders.

### 3.3 Social anxiety disorder

#### 3.3.1 PUFA-status

In individuals with social anxiety disorder (SAD), significantly reduced DHA and EPA concentrations, as well as an elevated omega-6/omega-3 ratio in erythrocytes, have been observed (see Table 4). In a study of patients with social anxiety compared to healthy controls, concentrations of most erythrocyte PUFA were lower (88). Comparing depressed individuals with and without comorbid anxiety disorders and a healthy control group, Liu et al. (89) also found the lowest EPA and DHA levels in patients with MDD and comorbid anxiety.

**Table 4:** Fatty acid composition in anxiety disorders and PTSD.

Study	Characteristics	DHA	EPA	n6/n3 ratio
Green et al. (88)	ERY; social anxiety disorder	↓	↓	↑ n6/n3
Liu et al. (89)	PLA; MDD with vs. without comorbid anxiety disorder	↓	↓	↑ AA/EPA
De Vries et al. (100)	ERY; PTSD	↓	=	
Silva and Singer (99)	ERY; trauma resulting from an intensive care unit stay	↓	↓	↑ n6/n3

Empty fields: not reported; ↑ increased in patients; ↓ decreased in patients; = no significant difference; comparisons were—unless otherwise noted—conducted between patients and healthy controls.

DHA, docosahexaenoic acid, 22:6ω3; EPA, eicosapentaenoic acid, 20:5ω3; ERY, erythrocytes; PLA, plasma; MDD, major depressive disorder; PTSD, post-traumatic stress disorder; n6/n3, omega-6/omega-3 ratio.

#### 3.3.2 Therapeutic potential of supplementation with omega-3 PUFA on anxiety symptoms

To investigate the potentially anxiolytic effects of omega-3 fatty acids, supplementation studies have been conducted in individuals with anxiety symptoms. However, since no studies to date have included patients with a formal diagnosis of SAD, the findings cannot be directly generalized to this population. A meta-analysis by Su et al. (90) of 1,203 participants with omega-3 PUFA supplementation (mean age = 43.7 years) and 1,037 participants without supplementation (mean age = 40.6 years) suggests that supplementation (mean omega-3 PUFA dosage = 1,605.7 mg/day) may lead to a significant reduction in anxiety symptoms, particularly with dosages (≥2,000 mg/day) and in subgroups with specific clinical conditions. The included studies are composed of both healthy participants without psychiatric or somatic diagnoses (91, 92), as well as clinically at-risk groups, for example, individuals with anorexia nervosa (AN) (93), substance use disorder (94), obsessive-compulsive disorder (95), depression (96), or those recovering from acute myocardial infarction (97). Given that anxiety disorders and stress are associated with hyperactivity of the hypothalamic-pituitary-adrenal (HPA) axis, the anxiolytic effects of omega-3 fatty acids may be mediated through the normalization of HPA axis activity (98).

#### 3.3.3 Conclusion

Due to the limited and heterogeneous data, it remains unclear whether omega-3 deficiency is a cause or consequence of increased anxiety symptoms, or whether both are mediated by third variables. The observed inverse association between omega-3 status and anxiety symptoms may also reflect reduced dietary intake, for example, for social and economic reasons, among individuals with anxiety disorders. Omega-3 PUFA deficiency may interact synergistically with external stressors, increasing vulnerability to the development and progression of anxiety disorders. In contrast, a balanced long-chain omega-3 PUFA status may act as a resilience factor, potentially mitigating anxiety symptoms in individuals exposed to stress (89). Well-designed intervention studies, including RCTs specifically targeting SAD, are therefore needed to investigate the therapeutic potential of omega-3 supplementation in this population.

### 3.4 Post-traumatic stress disorder

#### 3.4.1 PUFA-status

Several studies have reported lower long-chain omega-3 PUFA levels and an increased omega-6/omega-3 ratio in individuals with PTSD (see Table 4) (99). A detailed analysis of fatty acid profiles in a cross-sectional study with 49 PTSD patients and 46 healthy controls, after adjustment for sociodemographic and dietary influences, showed lower DHA in PTSD patients compared to healthy controls (100). Although no significant group differences in EPA levels were found, other researchers have reported a negative association between EPA and both PTSD risk and symptom severity (101, 102).

#### 3.4.2 Preventive potential of supplementation with omega-3 PUFA in PTSD

A recent systematic review by Capple et al. (103), including six studies (three preclinical and three RCTs), reported beneficial effects of omega-3 PUFA on heterogenous outcome measures for PTSD-like behavioral outcomes in animal models. The three clinical RCTs employed secondary preventive designs initiated after trauma exposure (mean age = 39 years) and administered daily doses of 1,470–1,568 mg DHA and 147–157 mg EPA (103–106). None demonstrated a significant reduction in PTSD onset (103). However, a secondary analysis of one of these studies reported that higher erythrocyte EPA levels were associated with lower PTSD symptom severity in the intervention group (*p* = 0.001) (106, 107). Additionally, one of these trials reported a significant decrease in heart rate after 3 months in the omega-3 group compared to the control group, and another found a significant reduction of PTSD severity in women (104, 105). Furthermore, a study of 83,391 adults (mean age = 56.1 years) from the UK biobank identified a significant inverse association between omega-3 PUFA level and PTSD risk. An additional MR analysis in this cohort further suggested a potential causal link of PTSD with lower omega-3 PUFA levels (beta = −0.203, *p* = 2.12E^−05^), with subgroup analyses showing stronger protective effects in women (OR = 0.64, *p* = 3.59E^−07^; *p*-interaction = 0.010) (108).

#### 3.4.3 Conclusion

In summary, current evidence suggests that alterations in fatty acid profiles may be associated with the onset and progression of PTSD. However, the causal relationship remains unclear and warrants further well-controlled studies, including trials investigating both secondary preventive approaches and the therapeutic potential of omega-3 supplementation in patients with established PTSD.

### 3.5 Anorexia nervosa

#### 3.5.1 PUFA-status

Observational studies and a meta-analysis of seven clinical trials predominantly report lower concentrations of the omega-6 fatty acids LA and AA, alongside elevated or unchanged omega-3 levels in young women with AN (109) (see Table 5). This shift may result from selective dietary habits, such as increased consumption of omega-3-rich foods, coupled with the deliberate avoidance of animal-based foods providing omega-6 PUFA (110, 111). Additionally, Swenne et al. (112) showed significantly lower DHA and EPA levels in anorexic individuals with comorbid depression compared to non-depressed patients (mean age = 15.3 years). In an MR study by Nomura et al. (113) based on inverse variance weighted methods, none of the genetically predicted plasma PUFA levels was significantly associated with anorexia nervosa risk, arguing against a protective role of omega-3 fatty acids on anorexia risk.

**Table 5:** Fatty acid composition in anorexia nervosa in women.

Study	Characteristics	DHA	EPA	LA	AA	n6/n3 ratio
Meta-analysis	Young women					
Satogami et al. (109)	PLA; ERY	=	↑ PLA		↓ ERY	↓ n6/n3 PLA
Studies from the meta-analysis						
Langan and Farrell (229)	PLA	↑		↓		
Sirinathsinghji and Mills (230)	PLA	↑				
Swenne et al. (115)	PLA; ERY	=	=	↓		=
Caspar-Bauguil et al. (231)	ERY	↓	↓		↓	↑ AA/EPA
Shih et al. (232)	PLA	↑	↑			↓ AA/EPA
Additional studies						
Holman et al. (233)	PLA	↓	↓		↓	
Zák et al. (234)	PLA	↓	=	↓	=	
Swenne and Rosling (114)	ERY	=	=		↓	=
Nguyen et al. (110)	PLA		↑			↓ n6/n3
Shimizu et al. (235)	PLA	=	↑	=	=	
Nguyen et al. (236)	PLA		↑	↑		=

Empty fields: not reported; ↑ increased in AN; ↓ decreased in AN; = no significant difference; comparisons were conducted between individuals with AN and healthy controls.

DHA, docosahexaenoic acid, 22:6ω3; EPA, eicosapentaenoic acid, 20:5ω3; LA, linoleic acid, 18:2ω6; AA, arachidonic acid, 20:4ω6; ERY, erythrocytes; PLA, plasma; n6/n3, omega-6/omega-3 ratio.

#### 3.5.2 The metabolic adaptation caused by negative energy balance affects PUFA metabolism

The activity of D6D, a key enzyme responsible for converting LA and ALA into their long-chain derivatives, has been reported to be downregulated under starvation conditions in a study of 24 adolescent girls with eating disorders (114). Because D6D shows a higher affinity for ALA and given the relative balance of omega-3 to omega-6 fatty acids, this may contribute to a suppression of omega-6 metabolism in patients with anorexia (114). Furthermore, studies indicate that lipid peroxidation is elevated during starvation, with LA being particularly susceptible to oxidation compared with omega-3 fatty acids, which could partly explain the observed reductions in LA and AA levels (115). Genetic factors may also play a role: Increased activity of soluble epoxide hydrolase (sEH), encoded by the EPHX2 gene, has been associated with omega-3 fatty acid accumulation in anorexia nervosa (109).

#### 3.5.3 Therapeutic potential of supplementation with omega-3 PUFA in anorexia nervosa

Given their metabolic properties, omega-3 PUFA have been investigated as therapeutic agents in anorexia nervosa. They are known to stimulate appetite and have been successfully used to support weight gain in cancer patients. Since AN typically begins in adolescence and is characterized by significant weight loss, systemic inflammation, and neurobiological changes in the brain, supplementation with long-chain omega-3 PUFA appears to be a promising therapeutic approach (111). Additionally, comorbid psychiatric disorders such as anxiety, depression, and obsessive-compulsive symptoms frequently occur in AN, for which some studies have reported beneficial effects of DHA or EPA supplementation (116).

A small case study by Ayton et al. (117) reported significant weight gain and mood improvement following a 3-month supplementation with 1 g/day EPA. Observed positive effects on weight (117, 118) and mood (117) may be attributable more to the appetite-stimulating properties of omega-3 fatty acids and the generally increased caloric intake during treatment (110). This evidence suggests that targeted, personalized PUFA therapy could be beneficial for the AN subgroup with psychiatric comorbidities (111). However, meta-analysis by Candido et al. (116), including five RCTs with a total of 144 participants (mean ages ranging from 14.7 to 33.5 years), found no significant effects of supplementation with omega-3 PUFA on eating disorder-specific symptoms, depressive mood, anxiety, or obsessive-compulsive symptoms compared to placebo. Additionally, Satogami et al. (109) reported in their meta-analysis of 379 patients (mean age = 18.3 years) compared with 164 controls (mean age = 23.5 years) that omega-3 supplementation did not improve disease severity or mood symptoms, but did improve body weight outcomes.

#### 3.5.4 Conclusion

Overall, individuals with anorexia nervosa exhibit altered PUFA profiles, characterized by reduced omega-6 levels and relatively preserved or elevated omega-3 levels. These profiles likely reflect both dietary patterns and metabolic adaptations to starvation. Although omega-3 PUFA have appetite-stimulating and neuroprotective properties and early case studies indicated potential benefits for mood and weight restoration, recent intervention studies provide no convincing evidence for a therapeutic effect. Further research is needed to determine if certain subgroups or treatment phases might benefit from targeted omega-3 supplementation.

### 3.6 Neuronal developmental disorders

#### 3.6.1 PUFA status

ADHD and ASD are multifactorial conditions that typically manifest in early childhood and often co-occur (119). Several studies have reported significantly reduced omega-3 fatty acid levels, particularly DHA, and an increased omega-6/omega-3 ratio in affected children. These findings suggest that an unbalanced nutritional intake, in combination with dysregulated PUFA metabolism, is a potential pathophysiological factor (27, 120–122).

Children with ADHD often exhibit pronounced deficiencies in DHA (*g* = −0.76; *p* < 0.001), EPA (*g* = −0.38, *p* < 0.001) and total n-3 (*g* = −0.58, *p* < 0.001), which can clinically manifest as increased thirst, dry skin, and dry eyes (25, 120). However, findings are inconsistent: two independent ADHD cohorts reported elevated EPA and DHA levels (123, 124), and Bell et al. (125, 126), as well as Bu et al. (127) found no significant PUFA abnormalities in children with ASD. The findings of an MR study by Wang et al. (128) utilizing data from the Psychiatric Genomics Consortium, including both childhood and adult ADHD, do not support causal links between PUFA status and ADHD.

#### 3.6.2 Preventive potential of supplementation with omega-3 PUFA in ADHD and ASD

PUFAs, as essential components of neuronal cell membranes, play a crucial role in key brain development processes (39). DHA is essential for optimal neural development—especially during the last trimester of pregnancy (32). A deficiency in DHA or an imbalance in the AA/DHA ratio during the fetal period or early childhood, therefore, may represent a modifiable risk factor for the development of ADHD and ASD (129). An imbalanced maternal diet, characterized by a high intake of omega-6 fatty acids combined with insufficient omega-3 consumption or abnormalities in PUFA metabolism, is associated with increased risks of preterm birth, low birth weight, and later developmental disorders. This is particularly relevant because a child's DHA levels during pregnancy and breastfeeding largely depend on maternal supply via the placenta and breast milk (39, 130).

To investigate the potential preventive effects of long-chain omega-3 fatty acids on ADHD and ASD, intervention studies have been conducted in pregnant and lactating women. Prenatal DHA supplementation was associated in some studies with improved cognitive development, attention, and executive functions, and it may act as a modifiable risk factor for neurodevelopmental disorders such as ASD and ADHD. Postnatal DHA maternal supply may also provide beneficial neuroprotective effects, further supporting its potential influence on the manifestation of ASD and ADHD (129–131). However, efficacy appears to depend heavily on the maternal nutritional baseline, particularly DHA levels and the omega-6/omega-3 ratio (131). The benefits of DHA are likely limited to cases of existing deficiency and imbalance. Furthermore, a sustained postnatal intake of sufficient amounts of long-chain omega-3 fatty acids is necessary to maintain these positive effects (119).

#### 3.6.3 ADHD

##### 3.6.3.1 Therapeutic potential of supplementation with omega-3 PUFA in ADHD

Meta-analytic evidence on PUFA supplementation in children and adolescents with ADHD shows modest beneficial effects on pre-existing ADHD symptoms, while prenatal and early-life DHA exposure appears to confer the most substantial neurodevelopmental protection (120, 121, 132). A meta-analysis of seven RCTs involving children and adolescents aged approximately 7–12 years with diagnosed ADHD (*n* = 534) showed that omega-3 PUFA supplementation (EPA: 180–1,200 mg/day, DHA: 120–1,000 mg/day) improved clinical symptoms of ADHD (*g* = 0.38, *p* < 0.0001), and enhanced attention-related cognitive performance (*g* = 1.09, *p* = 0.001) in three RCTs (*n* = 214) (120). Further evidence from a meta-analysis of 10 RCTs including 699 individuals with the clinical diagnosed of ADHD (mean age = 7–12 years) demonstrated that omega-3 PUFA supplementation (EPA: 0–750 mg/day; DHA: 0–480 mg/day) modestly improved ADHD symptoms [*SMD* = 0.31, 95% CI: (0.16–0.47), *z* = 4.04, *p* < 0.001]. Specifically, higher EPA doses were significantly associated with greater treatment efficacy [*g* = 0.36, 95% CI: (0.01–0.72), *t* = 2.34, *p* = 0.04, *R*^2^ = 0.38], supporting omega-3 supplementation as a potential adjunct to non-pharmacological interventions (132). In addition, a meta-analysis of 16 intervention studies including 1,408 children with ADHD (mean age = 9.7 years) found omega-3 PUFA supplementation (EPA: 0–1,373 mg/day; DHA: 0–1,140 mg/day) over a mean intervention period of 14.5 weeks modestly improved ADHD symptoms [*g* = 0.26, 95% CI: (0.15–0.37); *p* < 0.001]. Improvements were most consistent for hyperactivity, as reported by parents and teachers, while benefits in inattention were primarily observed by parent report (121).

An updated Cochrane review of 2021, including 37 randomized RCTs with more than 2,374 children and adolescents (mean age = 6–11 years) with diagnosed ADHD, examined the effects of PUFA supplementation, either alone or combined with co-interventions [e.g., methylphenidate (MPH), atomoxetine, physical training, and dietary supplements], compared with placebo or the same co-intervention alone. Supplements included omega-3 PUFA, omega-6 PUFA, or combined omega-3/omega-6 PUFA, administered for 2 weeks to 6 months. Evidence from 16 trials (1,166 participants) assessing parent-rated ADHD symptoms showed high-certainty evidence of no effect on total ADHD symptoms [SMD: −0.08, 95% CI: (−0.24 to 0.07)], inattention [SMD: −0.01, 95% CI: (−0.20 to 0.17)], or hyperactivity/impulsivity [SMD: 0.09, 95% CI: (−0.04 to 0.23)] compared with placebo. Low-certainty evidence from three trials (191 participants) suggested that PUFA may modestly increase the likelihood of symptom improvement in the medium term [RR: 1.95, 95% CI: (1.47–2.60)], but effects were generally small. Overall, the review highlights the limited therapeutic efficacy of PUFA supplementation in children and adolescents with ADHD, though methodological variability, small sample sizes, heterogeneous dosages, and short follow-up periods were identified as limitations (133).

However, systematic reviews suggest a nuanced efficacy profile, indicating that certain subgroups, such as children with comorbid developmental disorders, low dietary PUFA intake, low baseline PUFA blood levels, or predominantly inattentive symptoms, may particularly benefit from supplementation with long-chain omega-3 PUFA (25, 134). Combinations of EPA, DHA, and small amounts of omega-6 fatty acids like gamma-linolenic acid (GLA; 18:3ω6) appear more effective than EPA-only formulations (25, 135). For children with primarily hyperactive-impulsive behavior, higher EPA doses (≥500 mg/day) may yield greater benefits (56, 120), while DHA also plays a critical role in modulating neuronal functions and should not be neglected (136). Additionally, adjunctive PUFA supplementation may mitigate the adverse effects of MPH and reduce the required MPH dose, making combined therapy a promising option (137).

##### 3.6.3.2 Conclusion

Early or preventive intake of DHA during development appears to have stronger neuroprotective benefits. On the other hand, meta-analytic and systematic evidence shows that omega-3 PUFA supplementation in children and adolescents with diagnosed ADHD achieves only minor to moderate effects on clinical symptoms and attention performance. Higher doses of EPA may slightly increase efficacy, especially as a complementary treatment to non-pharmacological interventions. Overall, the therapeutic effect of PUFA in existing cases of ADHD is limited, while methodological heterogeneity, short study duration, and variable dosages reduce the evidence.

#### 3.6.4 Autism spectrum disorder

##### 3.6.4.1 Therapeutic potential of supplementation with omega-3 PUFA in ASD

There remains a lack of high-quality studies and robust evidence regarding the efficacy of supplementation with long-chain omega-3 PUFA in ASD, while existing research findings being heterogeneous and inconclusive (135, 138). Effect sizes appear to depend on the specific symptom clusters assessed and the diagnostic tools used, as highlighted by multiple systematic reviews (27, 56, 61, 135, 138, 139). In a meta-analysis of four RCTs including 107 individuals aged 3–28 years, Mazahery et al. (122) reported that omega-3 PUFA supplementation (EPA: 0.7–0.84 g/day; DHA: 0.24–0.70 g/day) over 6–16 weeks compared with placebo resulted in small improvements in social interaction [MD = −1.96, 95% CI: (−3.50 to −0.34); *p* = 0.02; *I*^2^ = 0%] and repetitive, restricted behaviors [MD = −1.08, 95% CI: (−2.17 to −0.01); *p* = 0.05; *I*^2^ = 0%], but showed no significant effects on communication, hyperactivity, or irritability. Completing this, a broader systematic review of 20 RCTs in 991 patients aged 2–40 years found that omega-3 PUFA supplementation over 6 weeks to 6 months had too weak an effect on core ASD symptoms to conclude meaningful improvement compared with placebo (138).

##### 3.6.4.2 Conclusion

While omega-3 PUFA supplementation may offer minor benefits for certain behavioral domains, current evidence does not support it as an effective intervention for core ASD symptoms, and further high-quality, well-powered studies are needed to clarify its therapeutic potential for autism.

### 3.7 Alzheimer's disease

Several mental illnesses across the life span (schizophrenia, affective and anxiety disorders, PTSDs and neurodevelopment disorders, and others) are risk factors for AD (140, 141). Factors such as chronic stress and the resulting altered HPA axis activity, increased neuroinflammation, oxidative stress, and mitochondrial dysfunction, reduced cognitive reserve, and other somatic comorbidities contribute to the development of AD as a consequence of mental illness. In addition, long-chain omega-3 PUFA deficiency could serve as a mediator between mental illness and AD. Within this framework, DHA emerges as a key factor in brain aging due to its neuroprotective effects: It contributes to stabilizing cell membranes, promotes membrane fluidity, protects neurons from oxidative stress, supports synaptic plasticity and may possibly facilitate the breakdown of beta-amyloid (142).

#### 3.7.1 PUFA status

A meta-analysis by Hosseini et al. (143) shows that older adults with mild cognitive impairment (MCI) or AD have significantly lower plasma DHA concentrations than age-matched controls. While PUFA alterations in MCI were primarily limited to DHA, patients with manifest AD exhibited deficits in almost all analyzed PUFAs. In contrast, a postmortem analysis of various brain regions from deceased individuals with and without AD found no significant reduction in DHA levels in brain tissue (144).

#### 3.7.2 Preventive potential of supplementation with omega-3 PUFA in Alzheimer's disease

Supplementation of omega-3 PUFA has been increasingly investigated for its preventive potential against cognitive decline and AD. Emerging evidence suggests that early and continuous intake of DHA, particularly when combined with physical activity and a balanced diet (142), could slow cognitive decline and delay the progression from MCI to manifest AD (145, 146). Using longitudinal data from 1,135 participants without dementia (mean age = 73 years) over 6 years, Wei et al. (145) demonstrated that omega-3 PUFA supplementation reduced the risk of AD [HR: 0.73, 95% CI: (0.55, 0.97); *p* = 0.029] compared to non-users of omega-3 fatty acids. Furthermore, long-term users had a 64% lower AD risk [HR: 0.36, 95% CI: (0.18, 0.72); *p* = 0.004] compared with non-users. Consistent with these findings, a systematic review including eight observational studies and 25 RCTs in adults aged ≥50 years, observational studies consistently suggested that higher dietary or supplementary intake of omega-3 PUFA (EPA and DHA) was associated with a lower risk of cognitive decline and future AD. Among included RCTs, those specifically investigating preventive effects in healthy older adults or individuals with MCI, mean participant ages ranged from 50 to 76 years, with supplementation doses of 160–2,000 mg/day DHA and 120–1,320 mg/day EPA, administered over periods of 3–60 months. These interventions were associated with improvements in short-term and working memory, delayed verbal recall, hippocampal volume, and cerebral perfusion, indicating that the cognitive benefits of omega-3 PUFA could be most effective when administered early, prior to or in the initial stages of cognitive decline (146). Overall, individuals with MCI or very mild AD seem to particularly benefit from increased DHA intake (147).

#### 3.7.3 Therapeutic potential of supplementation with omega-3 PUFA in Alzheimer's disease

Despite these promising possible preventive use, meta-analyses and reviews on supplementation with long-chain omega-3 PUFA in established AD have so far not shown significant improvements in cognitive functions or other Alzheimer's symptoms (146, 147). Kalamara et al. (147) included five RCTs in their systematic review (and four in the quantitative synthesis) comprising 702 patients with AD (376 receiving omega-3 PUFA and 326 placebo), with a mean age ranging from 72.6 to 78.95 years. Daily omega-3 supplementation ranged from 150 to 1,600 mg EPA and 350 to 2,000 mg of DHA over an intervention period of 6–24 months. The meta-analysis demonstrated no significant effect on cognitive performance, as assessed by the AD Assessment Scale-Cognitive Subscale [mean difference: 1.37, 95% CI: (0.00–2.73); *I*^2^ = 35%, *p* = 0.17; *z* = 1.96, *p* = 0.05]. In line with these findings, 10 RCTs involving patients with established AD with mean ages ranging from 70 to 76 years, supplementation with omega-3 PUFA with doses from 675 to 2,000 mg/day DHA and 300–975 mg/day EPA over periods of 3–24 months, largely did not improve cognitive outcomes as measured by AD Assessment Scale-Cognitive Subscale, Mini-Mental State Examination, and Clinical Dementia Rating, or relevant biomarkers (146). In advanced stages of the disease, structural damage and metabolic dysfunctions are usually so severe that, presumably as a consequence, supplementation is likely ineffective (148). Ebright et al. (142) provided a mechanistic explanation for the differences observed between preventive and therapeutic omega-3 PUFA studies. They note that age, lifestyle and environmental factors, genetic apolipoprotein-E-ε4-allele (APOE4) status, baseline omega-3/omega-6 intake, and disease stage all influence the brain's response to supplementation. In particular, APOE4 carriers seem to be more susceptible to blood–brain barrier dysfunction, oxidative stress, neuroinflammation, and impaired fatty acid metabolism, which reduce DHA uptake in the brain as AD progresses.

#### 3.7.4 Conclusion

Consequently, omega-3 PUFA supplementation seems to be most promising when implemented in at-risk individuals or in early stages of the disease, prior to substantial neuronal loss, whereas during established AD, alternative strategies targeting neuroinflammation or PUFA metabolism may be required (142, 149).

## 4 Discussion

### 4.1 Mechanisms of action of omega-3 PUFA

The findings presented in the preceding sections suggest that omega-3 fatty acids, particularly EPA and DHA, influence neurobiological processes in multiple ways due to their structural and functional properties. Their effects are based on the interplay of several molecular mechanisms, which are summarized below (see Figure 4).

DHA is an essential component of neuronal cell membranes and contributes significantly to membrane fluidity due to its high number of double bonds. This fluidity, in turn, influences lipid-protein interactions, such as those with membrane-bound receptors and transporters, thereby promoting synaptic communication (5). Thus, a deficiency of omega-3 fatty acids reduces membrane fluidity, thereby decreasing the efficiency of neuronal signal transduction, which could explain neurobiological correlates of mental illnesses. Additionally, EPA and DHA support the expression of brain-derived neurotrophic factor (BDNF), a key regulator of neurogenesis, synaptic plasticity, and long-term potentiation (LTP), a mechanism associated with learning and memory. A reduction in BDNF caused by oxidative stress can be counteracted by omega-3 PUFA, thereby enhancing synaptogenesis, neurogenesis, and neuroplasticity (150, 151). The anti-neurodegenerative effects of omega-3 PUFA are thus partly based on their ability to promote neurogenesis, BDNF synthesis, and synaptic plasticity (73).

Omega-3 fatty acids possess inflammation-modulating properties (20). EPA and DHA compete with omega-6 fatty acids such as LA and AA for the same enzymes during their synthesis, thereby inhibiting the production of pro-inflammatory eicosanoids derived from AA while simultaneously promoting the formation of anti-inflammatory eicosanoids and SPMs (5, 20). Furthermore, omega-3 fatty acids exert their inflammation-modulating effects through GPR120 and PPAR receptors. Activation of these receptors leads, among other effects, to a reduction in the activity of nuclear factor-kappa B (NFκB). This is a central transcription factor for pro-inflammatory cytokines, increasing the formation of anti-inflammatory transcription factors such as PPARγ (152, 153). Additionally, omega-3 metabolites can reduce oxidative stress and inflammatory processes (150).

Mental disorders are often associated with hyperactivity of the HPA axis and excessive cortisol secretion. This can lead to neuronal damage and increased stress reactivity. Omega-3 fatty acids modulate the HPA axis by influencing the secretion of corticotropin-releasing hormone (CRH) as well as the sensitivity of glucocorticoid receptors, thereby attenuating the stress response (5, 39, 73).

EPA and DHA exert regulatory effects on various neurotransmitter systems, particularly serotonin and dopamine, which are frequently dysregulated in psychiatric disorders (73). Furthermore, they can enhance the integrity of the blood-brain barrier, thereby limiting the passage of potentially neurotoxic substances into the brain and counteracting neuroinflammatory processes (154). Improved glucose uptake under the influence of DHA has also been reported (152). Omega-3 fatty acids additionally modulate the expression of genes involved in inflammatory processes and lipid metabolism (155). Their inhibitory effect on amyloid-β production may confer protection against neurodegenerative diseases such as AD (39).

Another significant mechanism concerns the gut microbiome, which is often dysregulated in stress-associated psychiatric disorders. Omega-3 fatty acids can positively influence the diversity and functionality of the intestinal microbiota—with potential effects on gut-brain communication and immune homeostasis (39).

### 4.2 Fatty acid status in mental disorders

In the majority of studies on the investigated mental disorders, differences in fatty acid profiles were found compared to healthy control groups (see Tables 1–5). In particular, lower EPA and DHA levels, as well as an increased omega-6/omega-3 ratio, appear to be associated with a higher risk for various mental illnesses. At the same time, both transdiagnostically and within individual disorders, the findings are heterogeneous. These inconsistencies may be attributable to methodological differences, the presence of confounding variables, and interindividual biological variability (5).

**Figure 3:**
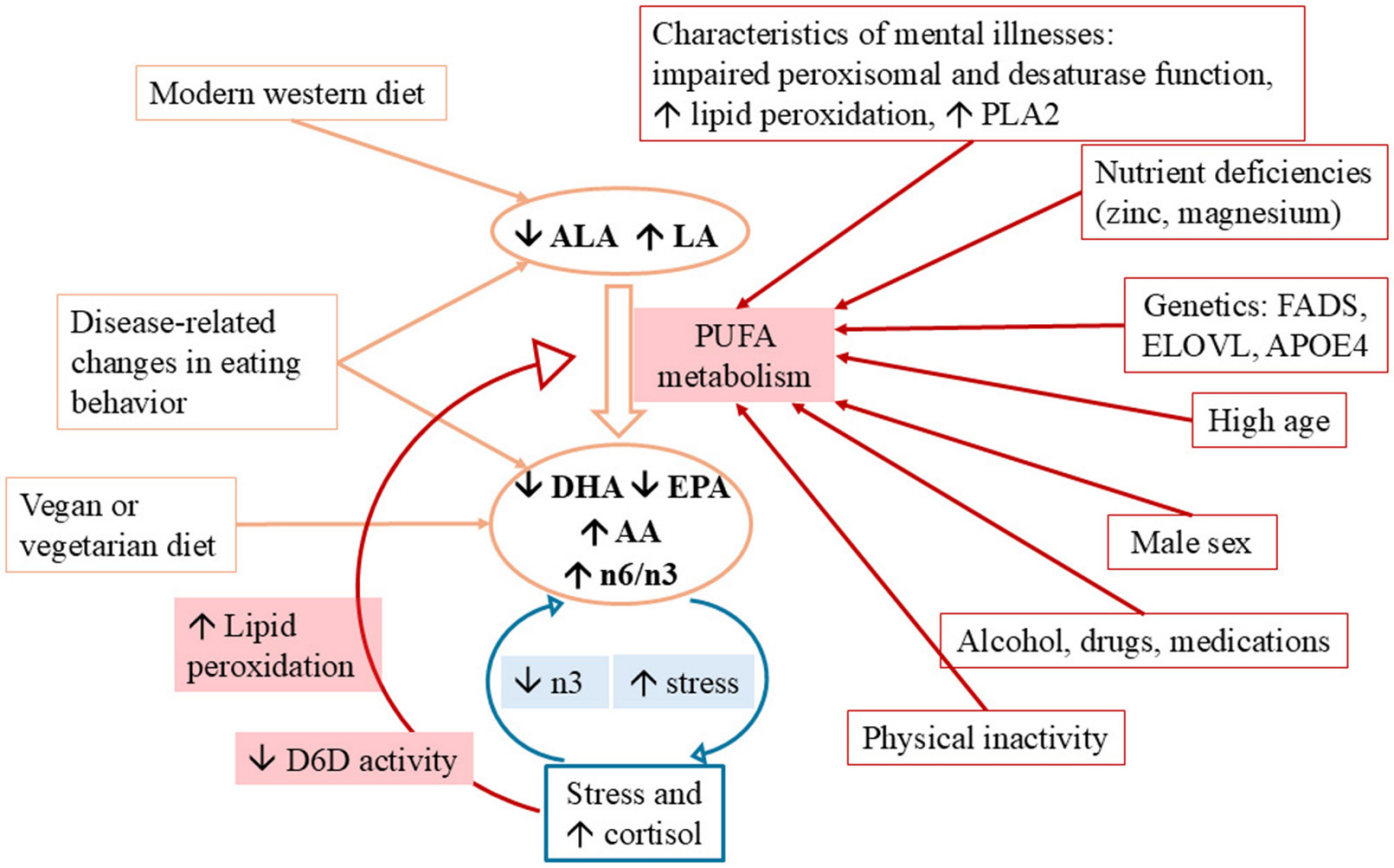
Possible causes of PUFA abnormalities. LA, linoleic acid, 18:2ω6; ALA, alpha-linolenacid, 18:3ω3; DHA, docosahexaenoic acid, 22:6ω3; EPA, eicosapentaenoic acid, 20:5ω3; AA, arachidonic acid, 20:4ω6; n6/n3, omega-6/omega-3 ratio; PLA2, phospholipase A2; D6D, delta-6-desaturase; n3, omega 3. The figure illustrates how various genetic, dietary, biological, and lifestyle factors affect PUFA metabolism and contribute to an increased n6/n3 ratio. A modern Western diet rich in LA and low in omega-3 precursors (ALA) shifts the balance toward AA and pro-inflammatory pathways. Additional influences include nutrient deficiencies, stress, physical inactivity, disease-related eating changes, and genetic variants (FADS, ELOVL, APOE4). A disturbed fatty acid profile may further promote oxidative stress and inflammation, particularly relevant in mental disorders, where stress and PUFA imbalance can form a self-reinforcing cycle.

The observed abnormalities in fatty acid profiles are likely multifactorial in origin, resulting from a complex interplay of genetic, biological, and environmental influences (see Figure 3). Nutrition plays a central role: the drastically altered nutrient composition due to industrialization, especially the sharp increase in LA intake to 6%−8% of daily energy, has led to an excess of omega-6 fatty acids and a relative deficiency in omega-3 PUFAs. This imbalance promotes skewed PUFA synthesis in favor of pro-inflammatory omega-6 metabolites (13). In addition, excessive consumption of processed carbohydrates and fats may impair omega-3 metabolism (156). Certain dietary patterns, such as vegan or vegetarian diets, may also contribute to omega-3 deficiencies if marine sources like fish are not adequately included (149).

Beyond nutrient intake alone, other factors also influence PUFA metabolism. These include zinc and magnesium status, as well as the intake of trans fatty acids, which can stimulate or inhibit desaturase activity, respectively (14, 123). For example, magnesium deficiency may lead to reduced activity of magnesium-dependent D6D enzymes, adversely affecting fatty acid composition (157). Additionally, illness-related changes in eating behavior—such as appetite loss or selective diets commonly seen in psychiatric disorders—can negatively impact omega-3 status (42, 139).

At the genetic level, polymorphisms in the FADS and ELOVL genes influence the conversion efficiency of essential PUFAs (such as LA and ALA) into their long-chain derivatives like AA, EPA, DPA, and DHA. For instance, a FADS haplotype associated with increased conversion capacity is more common in African populations, while variants linked to less efficient PUFA synthesis are prevalent in individuals of European descent. When combined with a diet high in LA, this may lead to a relative omega-3 deficiency (13). Additionally, APOE4 polymorphisms have been linked to increased DHA oxidation and disrupted fatty acid homeostasis (142, 158).

Additional individual factors such as age, sex, physical activity, alcohol and drug use, and medication intake also influence fatty acid status (3, 42, 159, 160). With increasing age, PUFA levels in the brain decline, which appears to be associated with a reduction in gray matter volume (161). Women generally show higher DHA levels than men, likely due to estrogen-induced stimulation of DHA synthesis—supporting the relevance of sex-specific analyses (162). Variations in sex distribution across study populations may contribute to inconsistent findings. Furthermore, intersex and transgender individuals are typically excluded from studies, which further limits the generalizability of the results to the broader population.

An unfavorable omega-6/omega-3 ratio and a deficiency in omega-3 fatty acids are also associated with increased inflammatory activity and oxidative stress—pathophysiological processes commonly observed in psychiatric disorders (5, 42). Furthermore, the relationship between stress and fatty acid status appears to be bidirectional, and it may manifest as a vicious cycle: psychosocial stress raises cortisol secretion, which can lower omega-3 levels, while pre-existing omega-3 deficiency may amplify stress reactivity and cortisol production (5, 163). Stressors also alter fatty acid metabolism by inhibiting desaturase activity, promoting lipid peroxidation, and enhancing the production of pro-inflammatory cytokines (164). Alterations in fatty acid composition observed in psychiatric disorders may additionally result from impaired peroxisomal function, defective desaturase enzymes, increased PLA2 activity, or heightened lipid peroxidation—mechanisms that are frequently associated with mental illness (164–166).

Ultimately, the question of causality remains unresolved: Are the altered fatty acid levels etiologically relevant to the development of psychiatric disorders, or are they rather a consequence of the disease? This central issue calls for further longitudinal and MR studies to clarify the direction of the association (167).

### 4.3 Explanations for the heterogeneity in RCT outcomes

The results of RCTs investigating the use of omega-3 PUFA in mental disorders are heterogeneous due to numerous methodological limitations and currently do not allow for definitive clinical recommendations (39). Furthermore, there is a lack of studies that have measured actual omega-3 fatty acid deficiencies in the blood, substituted them with individually adjusted doses, and re-checked the blood values. Many studies are characterized by small sample sizes, biases, and low effect sizes (167). Additionally, a potential publication bias exists, as a substantial proportion of trials are funded by supplement manufacturers and tend to report predominantly positive findings (133). Blinding efficacy may be compromised by the distinctive taste of fish oil. Furthermore, the placebo substances used—such as olive oil or other plant-based oils—carry the risk of methodological bias, as they may exert independent psychoactive effects (58).

Heterogeneous study designs and inconsistent analyses hinder clear conclusions. Positive effects mainly appear in RCTs with well-defined, severe cases, and adjunctive omega-3 therapy seems to be more effective than supplementation alone (58, 72). Effectiveness also depends on supplement quality, composition, dose, timing, and duration. To maximize bioavailability, omega-3 PUFA should be taken with fatty meals and protected from oxidation by antioxidants and proper storage (72, 73). Both active and placebo products may contain bioactive compounds that affect results (58, 73). In addition, the significant influence of the background diet should be taken into account. Ideally, this should be standardized using holistic nutritional concepts in order to minimize its influence.

Although DHA is the predominant fatty acid in gray matter and deficiencies are frequently observed in psychiatric disorders, EPA is attributed to greater clinical efficacy (5). Supplements vary considerably in their EPA/DHA ratios. An EPA-dominant formulation with a 2:1 ratio and a daily dose of 1–2 g demonstrated protective effects in multiple studies (72, 73). Negative findings in other studies may be attributable to suboptimal dosing or unfavorable supplement composition. Treatment duration and timing are also critical, as the organism requires time to respond to supplementation, and deficits identified early are often more effectively corrected (54, 158).

Moreover, numerous individual factors act as confounders: age, sex, genetics, baseline nutrient status, background diet, medication use, physical activity, lifestyle factors, compliance, comorbidities, as well as the type and severity of the disorder influence the individual response to omega-3 supplementation. Many RCTs do not control for or adequately report these variables, limiting comparability (99). It must also be acknowledged that omega-3 fatty acids represent merely a single element within the framework of a nutritionally optimal and balanced dietary regimen. To counteract this, it is essential to monitor bioavailability and compliance with omega-3 PUFA interventions by regularly measuring concentrations in plasma or erythrocyte lipids.

This high interindividual variability underscores the need for personalized medicine tailored to individual PUFA status, lifestyle and nutritional preferences, and the occurrence of comorbidities.

## 5 Implications and future directions

An omega-3 fatty acid deficiency as well as an imbalanced omega-6/omega-3 ratio can be considered potential, modifiable risk factors in the multifactorial etiology of mental disorders. Such fatty acid patterns have been particularly observed in schizophrenia and affective disorders. Similar alterations have also been noted in anxiety disorders, PTSD, ADHD, ASD, and AD. In contrast, previous studies on anorexia nervosa show no evidence of omega-3 deficiency.

**Figure 4:**
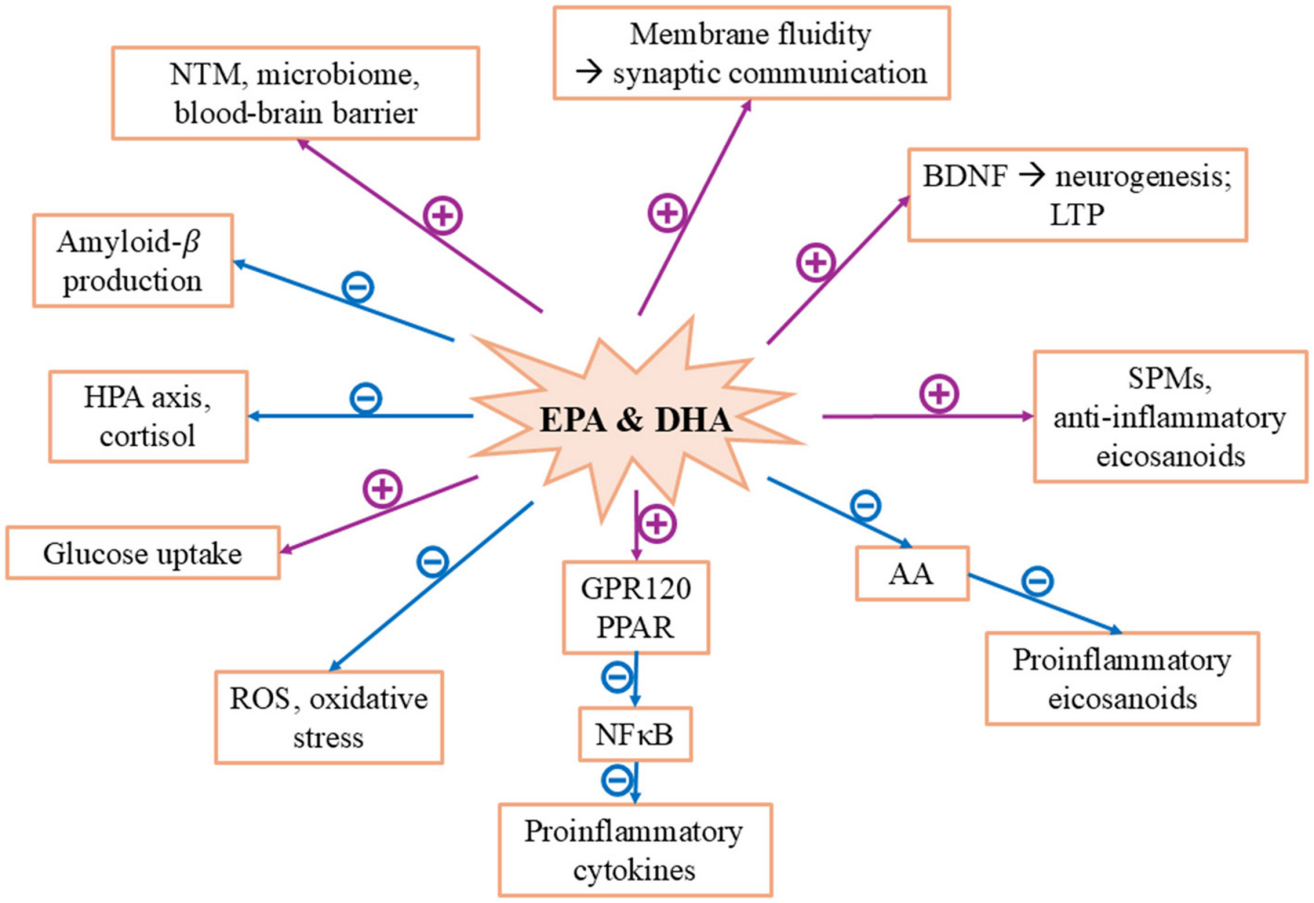
Mechanisms of action of EPA and DHA. NTM, neurotransmitter; BDNF, brain-derived neurotrophic factor; LTP, long-term potentiation; HPA, hypothalamicpituitaryadrenal; ROS, reactive oxygen species; NFκB, nuclear factor-kappa B; AA, arachidonic acid, 20:4ω6; SPMs, specialized pro-resolving mediators; GPR120, G-protein coupled receptor 120; PPAR, Peroxisome Proliferator-Activated Receptor. Omega-3 fatty acids influence brain function via multiple pathways: DHA enhances membrane fluidity and synaptic signaling; both EPA and DHA promote BDNF expression, supporting neurogenesis and plasticity. They exert anti-inflammatory effects by competing with omega-6 fatty acids, promoting pro-resolving mediators, and activating GPR120 and PPAR receptors. Their antioxidant properties reduce oxidative stress. Omega-3s modulate the HPA axis, dampening cortisol release and stress reactivity. They influence neurotransmitter signaling, support bloodbrain barrier integrity, enhance cerebral glucose uptake, and may reduce amyloid-β accumulation. Additionally, they promote gut microbiome diversity, potentially improving gutbrain communication.

Given the complex interactions between mental disorders and fatty acid metabolism, as described above, changes in fatty acid profiles cannot be considered merely epiphenomenal, meaning they are not merely consequences of mental illness or associated lifestyle factors, such as poor nutrition, lack of exercise, or medication. Instead, the available evidence suggests that changes in omega-3 fatty acid status could play a predisposing or causal role in the development of mental disorders. This can be explained by the assumptions shown in Figure 4. In summary, a deficiency in omega-3 fatty acids contributes to immune and brain metabolic imbalances via various interacting and interrelated mechanisms, including mitochondrial dysfunction, oxidative stress, inflammatory metabolism, and HPA axis disruption. These pathophysiological relationships may also shed light on the comorbidity of mental disorders with numerous physical illnesses such as cardiovascular diseases and type 2 diabetes mellitus (3).

In order to confirm these pathophysiological causality assumptions, further methodologically rigorous studies, such as randomized controlled intervention trials, are needed. These studies should assess participants' baseline fatty acid status, dietary intake, and preferences; monitor changes in fatty acid profiles during the intervention; and include larger, more homogeneous samples with longer follow-up periods. Standardization of study designs would also be necessary to facilitate future meta-analyses and systematic comparisons (73).

In addition to randomized controlled studies, MR studies could also contribute to our understanding. These studies use genetic variants as natural “random experiments” to investigate causal associations between risk factors and diseases. Confounding and reverse causality are minimized, as genes are randomly distributed and are not influenced by environmental factors or disease. This enables them to provide more robust evidence of causality than classical observational studies. They are therefore particularly well-suited to investigate the relationship between dietary factors, such as omega-3 fatty acids, and psychopathology. For example, a recent MR analysis by Wu et al. (168) found no significant association between fish oil intake and mental disorders such as anxiety, bipolar disorder, dementia, depression, mood disorders, PTSD and schizophrenia. In contrast, another MR study in genetic colocalization analyses identified the predictive function of omega-3 fatty acids for reducing the likelihood of major depressive disorder. The greatest effects were observed for EPA (85).

Given the multitude of factors influencing PUFA metabolism, future studies on fatty acid composition and supplementation should be designed more comprehensively. This includes systematically assessing not only dietary omega-3 intake but also micronutrient status (e.g., magnesium and zinc) and the intake of other dietary components that interact with PUFA metabolism. Additionally, genetic predispositions, disease-related changes, and individual lifestyle factors (e.g., vegan, vegetarian, and mixed diet) should be incorporated into the analysis. Moreover, further studies should also consider the relative contribution of dietary fat vs. carbohydrate intake, as emerging evidence suggests that ketogenic or ketone-enhancing dietary patterns may be beneficial in psychiatric and neurological conditions. There is growing evidence that the ketogenic diet may reduce inflammatory processes and reactive oxygen species, support the restoration of neuronal myelin and promote mitochondrial biogenesis (169, 170). Such an integrative approach is currently lacking but could be crucial for explaining the existing heterogeneity in study findings.

Irrespective of its causal relationship to mental illness, the potential role of PUFA composition as a biomarker is a promising avenue for future research. This development could facilitate the development of personalized therapeutic interventions, as evidenced by recent studies (5).

Furthermore, omega-3 fatty acids offer promising therapeutic potential in the prevention and treatment of psychiatric disorders. They possess health-promoting properties and are associated with relatively low costs, high tolerability, and good compliance. Studies report effect sizes comparable to those of conventional antidepressants (5). According to the European Food Safety Authority, a daily intake of up to 5 g is considered safe, with a recommended omega-3 index of less than 16%. According to the US-American Food and Drug Administration, up to 3 g/day are safe (149). Adverse effects are generally mild and include gastrointestinal discomfort, burping, or skin irritations (72). However, higher dosages have been linked to an increased risk of bleeding or atrial fibrillation (171). According to a Drug Safety Information from 2023, omega-3 supplementation is associated with a dose-dependent increased risk of atrial fibrillation in patients with established cardiovascular disease or cardiovascular risk factors. Therefore, supplementation in this population is only recommended with caution and under medical supervision (172). A few isolated studies even reported a slight increase in depressive symptoms among healthy older adults and individuals with PTSD following supplementation of omega-3 fatty acids from fish oil (173, 174).

Despite their widespread availability, omega-3 supplements should not be taken uncritically. These are bioactive substances that, unlike pharmaceuticals, are not subject to mandatory efficacy or safety testing. Low product quality, insufficient dosages, or unfavorable fatty acid ratios and the occurrence of oxidation products due to unfavorable conditions during the manufacture of the supplements may impair effectiveness or even cause adverse effects. Furthermore, there is a risk that affected individuals might neglect important lifestyle changes or replace established therapies due to the hype surrounding long-chain omega-3 PUFA (167). Moreover, the optimal dosage, formulation, timing, and duration of supplementation depend largely on individual factors.

It is therefore essential that both healthcare professionals and the general public are informed about the potential benefits of adequate omega-3 PUFA intake as well as the limitations and risks of supplementation. Presently, there remains an absence of prepared informational materials from official information resources that are independent of private companies. In addition, professional training for responsible professionals (psychiatrists, psychosomatic physicians, psychotherapists, nurses, etc.) regarding the current evidence base is required. Patients diagnosed with mental illness often do not consume balanced diets rich in omega-3 fatty acids. Thus, a systematic assessment of their current diet is necessary to identify deficiencies and counteract, for example, due to supplementation with long-chain omega-3 PUFA under medical supervision, including laboratory monitoring.

Overall, the existing findings suggest that insufficient omega-3 fatty acid supply cannot be regarded as a sole cause but rather as a significant etiological contributing factor in mental disorders. Therefore, its role should be further investigated and clinically considered within the framework of an integrative model of mental illnesses, both preventively and therapeutically.
